# K-Pax2: Bayesian identification of cluster-defining amino acid positions in large sequence datasets

**DOI:** 10.1099/mgen.0.000025

**Published:** 2015-07-15

**Authors:** Alberto Pessia, Yonatan Grad, Sarah Cobey, Juha Santeri Puranen, Jukka Corander

**Affiliations:** 1 Department of Mathematics and Statistics, University of Helsinki, Finland; 2 Department of Immunology and Infectious Diseases, Harvard T.H. Chan School of Public Health, Boston, Massachusetts, USA; 3 Division of Infectious Diseases, Department of Medicine, Brigham and Women's Hospital, Harvard Medical School, Boston, Massachusetts, USA; 4 Department of Ecology and Evolution, University of Chicago, Chicago, Illinois, USA; 5 Department of Biosciences, Åbo Akademi University, Turku, Finland

**Keywords:** data clustering, protein evolution, sequence analysis

## Abstract

The recent growth in publicly available sequence data has introduced new opportunities for studying microbial evolution and spread. Because the pace of sequence accumulation tends to exceed the pace of experimental studies of protein function and the roles of individual amino acids, statistical tools to identify meaningful patterns in protein diversity are essential. Large sequence alignments from fast-evolving micro-organisms are particularly challenging to dissect using standard tools from phylogenetics and multivariate statistics because biologically relevant functional signals are easily masked by neutral variation and noise. To meet this need, a novel computational method is introduced that is easily executed in parallel using a cluster environment and can handle thousands of sequences with minimal subjective input from the user. The usefulness of this kind of machine learning is demonstrated by applying it to nearly 5000 haemagglutinin sequences of influenza A/H3N2.Antigenic and 3D structural mapping of the results show that the method can recover the major jumps in antigenic phenotype that occurred between 1968 and 2013 and identify specific amino acids associated with these changes. The method is expected to provide a useful tool to uncover patterns of protein evolution.

## Data Summary

1. Supplementary Text S1 has been deposited in figshare: 10.6084/m9.figshare.1334296
2. Supplementary Tables S1–S3 have been deposited in figshare: 10.6084/m9.figshare.1334294
3. Supplementary Fig. S1 has been deposited in figshare: 10.6084/m9.figshare.1334297
4. Supplementary Video S1 has been deposited in figshare: 10.6084/m9.figshare.1334293

## Impact Statement

Large sequence databases have introduced new opportunities to explore patterns of microbial evolution. This paper introduces the first fast model-based machine learning method targeted to identify genomic positions that are likely to display non-synonymous variation due to selection pressure. The method is widely applicable to aid in generation of hypotheses for experimental work and to pinpoint plausible candidates for further study and data acquisition. Results on influenza A/H3N2 highlight the potential to significantly advance the process towards understanding the mechanisms linked to the success of major pathogens.

## Introduction

The growth in microbial genome sequence data, driven by decreasing sequencing costs and the integration of sequencing into routine clinical microbiology (Köser *et al.*, 2012; Reuter *et al.*, 2013), has begun to revolutionize our understanding of microbial evolution and spread. However, the pace of sequence accumulation generally exceeds the pace of experimental studies of protein function. This relationship holds not only for recently emerged pathogens (Cotten *et al.*, 2013; Gire *et al.*, 2014), but also for intensively studied pathogens, such as influenza (Gong & Bloom, 2014; Worobey *et al.*, 2014). Tools to analyse such large datasets and provide targeted guidance in inferring phenotypically meaningful groups can therefore be useful to identify amino acid sites and proteins that play critical roles in pathogen biology and evolution. These sites are potential targets for diagnostics, therapeutics and vaccines.

Large sequence alignments are challenging to dissect using standard tools from phylogenetics and multivariate statistics. When the datasets comprise hundreds to thousands of sequences, trees become increasingly crowded and identifying meaningful information is difficult. In contrast, basic statistical procedures such as principal components analysis, hierarchical clustering or *k*-means (Hastie *et al.*, 2009) can provide a compressed view into the data with relative ease. However, the use of such unfocused methods for extracting information is problematic when the biologically relevant signals are masked by noise introduced due to sequencing errors or functionally neutral variation. This is the situation for fast-evolving organisms where many changes rapidly accumulate across proteins but only a subset of them actually show signs of selection.

Model-based statistical methods have a clear advantage over the generic approaches when the model is structured to infer biologically relevant information. For microbial proteins one important question is which isolates or strains constitute phenotypically distinct groups, distinguished by specific amino acids fixed by selection. It is also useful to know which positions and amino acids are probably directly under selection. Statistically, these questions correspond to the task of simultaneously clustering a protein sequence alignment in two ways, by the rows to identify the relevant groups of strains and by the columns to identify which amino acid positions define the clusters. As both the number of groups and the relevant sequence positions are often unknown, statistical inference is required. Bayesian modelling is particularly well suited for such model selection problems, as by specifying probabilistic prior information for the unknowns in the model, one can efficiently focus the search and avoid overfitting.

Previous studies (Aguas & Ferguson, 2013; Meroz *et al.*, 2011) have partially solved the above-discussed problem by supervised machine learning techniques. Within this related setting, genetic determinants are identified conditional on a known classification of the sequences. To our knowledge, no statistical machine learning method has yet addressed the problem of identifying most relevant sites and amino acids without knowing a priori how the sequences are grouped.

We introduce here a Bayesian method (K-Pax2) that can handle thousands of sequences with minimal subjective input from the user. Our approach is based on a two-way clustering model inspired by an earlier method (K-Pax) for clustering single protein sequence alignments from distant homologues to identify substructure within a protein superfamily (Marttinen *et al.*, 2006). Our current method possesses two significant improvements over the original K-Pax, one related to accuracy and the other to the technical specifications of the priors and model. These changes permit the method to be used to study a large number of closely related sequences as well as several proteins simultaneously. A useful feature of our model definition is that it enables an analytically obtainable Bayesian score of model fitness. This feature permits the use of parallel computation in model optimization, as the scores are directly comparable from independent optimization runs without approximation errors caused by, for example, Monte Carlo methods.

The haemagglutinin (HA) of influenza A/H3N2 possesses features that make it an ideal test case to demonstrate the function and applicability of K-Pax2 to large alignments. Thousands of A/H3N2 HA sequences are available in public databases (Bao *et al.*, 2008; Benson *et al.*, 2005; Bogner *et al.*, 2006; Squires *et al.*, 2012). In addition, the detailed structure and evolution of HA have been investigated by phylogenetic inference and direct experiments (Bedford *et al.*, 2014; Bizebard *et al.*, 1995; Fleury *et al.*, 1999; Knossow *et al.*, 2002; Koel *et al.*, 2013; Smith *et al.*, 2004; Suzuki, 2006; Wolf *et al.*, 2006).

HA is a homotrimeric integral membrane protein on the surface of the influenza virion and the primary target of the neutralizing immune response against influenza. HA binds sialic acid receptors on the surface of cells and, once bound, promotes viral entry by fusion of the viral envelope with the endosome membrane. The tertiary structure of HA indicates that there are two main domains: a variable globular head (HA1) that contains the sialic acid binding sites and a conserved stalk region (HA2) involved in membrane fusion (Skehel & Wiley, 2000).

Since its introduction in 1968, the A/H3N2 HA has undergone rapid evolution that is associated with short coalescent times, a ladder-like phylogeny and regular antigenic change (Bedford *et al.*, 2014; Fitch *et al.*, 1991; Smith *et al.*, 2004). The HA1 domain is the predominant site of influenza's antigenic evolution. Mutations in exposed epitopes demonstrate strong selective pressure to escape antibodies (Fitch *et al.*, 1991; Suzuki, 2006), and tend to predominate along the trunk of the phylogenetic tree. However, there is also evidence of positive selection at CD4+ and CD8+T-cell epitopes (Suzuki, 2006) and for the addition of *N*-linked glycosylation sites (Suzuki, 2011). Here, we use the K-Pax2 method to analyse thousands of influenza A/H3N2 HA sequences to evaluate the success of the algorithm in identifying amino acid positions known to play key roles in the function of HA.

## Theory and Implementation

### A two-way clustering model for identifying groups of viral strains under diversifying or directional selection

Let  denote a multiple sequence alignment of concatenated amino acid sequences for the coding regions extracted from *n* virus samples. Each alignment element thus belongs to the alphabet  representing the set of amino acids, including the gap symbol. The length of the aligned sequences is denoted by *L*. For the purpose of obtaining a model family and an inference algorithm that can efficiently capture signals of diversifying and directional selection from ***S***, we transform the multiple sequence alignment into an  binary matrix, where each column corresponds to an indicator variable of a particular element in  being observed at position *l*. Prior to any inference, all columns with exclusively zero elements are removed from the analysis because they are uninformative for the statistical model introduced here. The resulting binary matrix ***X*** is assumed to be of dimension *n* × *m*.

In a set notation, let  denote the collection of integer labels for the *n* virus strains. Let  denote an assignment of the *n* strains into *K* mutually disjoint non-empty clusters, where ***w***_*K*_ represents the set of labels of the units associated with cluster *k*. Formally, the *K* non-empty subsets  define a partition of the sequences such that  and , . In our model formulation each of the *K* clusters is assumed to correspond to a group of strains that has evolved under diversifying or directional selection pressure and consequently proliferated given the fitness improvements induced by non-synonymous changes that are of functional importance at the protein level. The sequence locations of such changes, the number of groups *K* and the explicit assignment of strains into the groups are all unknown parameters of our model to be inferred from the matrix ***X***.

Non-synonymous changes in viral strains that are free from diversifying selection pressure will fluctuate in frequency in the population due to drift, but they are not in general expected to be rapidly driven to fixation unless they are tightly linked to other sites that are under selection. We assume that the non-synonymous mutations that do not induce fitness changes will occur at a constant rate throughout the population. This can be translated into the statistical approximation that for the *n* sampled strains, functional neutrality corresponds to a fixed probability of observing a particular residue in a given sequence position across all the *K* clusters:for all *i* ∈  ***w***_*k*_ and for all , *K*. Thus, from the clustering perspective, any column  in ***X*** is considered as ‘noise’ if the above probability is constant across groups. Conversely, we define a column *j* to represent a putative ‘selection signal’ if there are at least two groups for which the corresponding probability is different:for all *i* ∈  *w*_*k*_, and *i*′ ∈  ***w***_*k*′_ and for some *k* ≠  *k*′. Such signals are only putative, as random drift could still explain a difference in the residue composition between two clusters. In addition, more rigid probabilistic restrictions must be imposed on the model structure to ensure that the grouping ***W*** and the identities of the selected sites become jointly identifiable and convey a biologically meaningful extraction of information from the alignment ***S***. Note that residues that remain unchanged in the whole virus population over long periods of time ostensibly due to strict functional constraints on the protein structure are also uninformative for the purpose of identification of sequence clusters, as they correspond to fully conserved sites in the alignment ***S***.

Under relatively strong selection pressure, non-synonymous changes that are associated with an increase in fitness should rapidly rise in frequency, leading to the formation of a novel group of strains. Similar to the neutral changes considered previously, this assumption can be translated into a statistical approximation that implies that we expect for each cluster *k* at least a single column *j* to be present in ***X*** such thatThese residues are defined as ‘characteristic’ for cluster *k* and represent significant signals of selection. In summary, a site–amino acid pair (column of ***X***) can then be considered either noise (*h* = 1), a weak signal (*h* = 2) or a strong signal (*h* = 3). It can be further classified as of no particular status (*r* = 1) or characteristic (*r* = 2) for a cluster. Column classification can accordingly be encoded by a collection of binary variables *z*_*jhrk*_ attaining value 1 if and only if column  has property *h* (*h* = 1, 2, 3) with status *r* (*r* = 1, 2) in cluster , and attaining value 0 otherwise. Let the array ***Z*** represent the collection of binary variables *z*_*jhrk*_ over all the index values. The pair (***W***,***Z***) then contains all the main parameters of interest in our model. However, its full probabilistic characterization requires a set of additional nuisance parameters that are defined below.

### Likelihood function

Assuming conditional independence of the elements of ***X*** given both the main and the nuisance parameters of the model, we obtain the following expressions:where ***X***_*k*_ is the binary data matrix associated with cluster *k* of size *n*_*k*_, and subsequentlywhere ***x***_*kj*_ is the binary vector for cluster *k* at column *j*, while ***Z***_*jk*_ is a  binary matrix such that . Defining the columns as statistically independent may be interpreted as a very strong assumption. However, note that their stochastic nature is already addressed through the prior distribution. Concern could arise for phenomena such as hitchhiking, where sites could be genetically linked and thus present at similar frequencies. Such cases can be easily addressed when post-processing the results from model optimization.

We define the (prior) predictive probabilitywhere  is the Bernoulli distribution and  is the conjugate Beta distribution for its parameter , which is explicitly conditioned on the property and status of column  in cluster . All these Bernoulli parameters are nuisance parameters in the model, as their explicit values are not a target of inference. Hence, in accordance with standard conventions in Bayesian statistics, they are integrated out from the likelihood to obtain the marginal posterior distribution for the parameters of interest. Note that according to this formulation sequences belonging to the same cluster are not statistically independent, whereas sequences belonging to different groups are. For , standard Bayesian calculation (Bernardo & Smith, 2000) shows that equation 2 is equal to the ratio of Beta functionswhere  and  where  and  are the hyperparameters of the Beta distribution and  is the number of values equal to unity observed in cluster  at column . To simplify the notation, we denote the probability in equation 2 as . The likelihood function can now be compactly rewritten as

### Prior distributions

Let  be the joint prior distribution for the partition ***W*** and the column classification ***Z***. For computational simplicity, similar to Marttinen *et al.* (2006, 2009), we define the prior distribution for ***W*** as the uniform distribution for whichThere are alternative prior distributions for data partitions that directly penalize an increase in the number of clusters, such as a uniform distribution for the number of clusters *K* used in the hierBAPS software (Cheng *et al.*, 2013) or the Dirichlet process prior (Jain & Neal, 2007; Neal, 2000). However, because we use a strongly informative prior distribution for the parameters in ***Z***, which penalizes spurious clusters, the uniform prior on ***W*** does not lead to problems with overestimation of *K*, as illustrated for a related clustering model by Marttinen *et al.* (2009).

To define the conditional prior distribution for ***Z***, we follow a hierarchical approach. Let γ = (γ_1_, γ_2_, γ_3_)^*T*^ denote our prior probabilities for a column to represent noise, weak signal or strong signal, respectively. Then, γ_*h*_ ≥  0 and . Note that these properties are column-specific and they are not affected by any particular partition under consideration. Also, note that the array ***Z*** satisfiesfrom which we obtainand consequently *z*_*jh*.._/*K* can be interpreted as an indicator variable, taking value 1 if and only if column *j* has the property *h*. Assuming the columns to be stochastically independent from each other, motivated by the lack of any prior information about their relationships, we start by writingwhere ***Z***_*jh*_ is a  binary matrix satisfying equation 5. The matrix ***Z***_*jh*_ is then modelled by *K* independent multinomial distributionswhere ω_*hr*_ is the prior probability of observing status *r* when a column has property *h*. Inserting equation 7 into equation 6, we finally obtainwhere we used the equality .

### Posterior inference

By multiplying the right-hand side of equation 4 and equation 8, we obtain the joint posterior distribution of the main parameters up to a normalizing constantWe estimate the pair (***W***, ***Z***) using the mode of the posterior distributionwhich is equivalently obtained by maximizing the log posterior while ignoring the constant term:Let  represent the set of all the possible partitions of  and let  denote the set of all the possible classifications of the columns (conditional on the underlying partition). The cardinality of the parameter space is easily determined, as  is equal to the Bell number *B*_*n*_, whereas . For a discrete posterior distribution over a space of such high cardinality and complex topology, it is unlikely that any standard Markov chain Monte Carlo approach would be able to efficiently explore the distribution and estimate the mode using a reasonable amount of computational time. Therefore, we have developed a greedy optimization algorithm for fitting the model to a multiple sequence alignment. An advantage of the analytical tractability of the model is that any two model structures can be compared using the difference in log posterior, and hence estimates from multiple independent parallel or sequential algorithm runs can be ranked in a straightforward manner. Similarly, posterior uncertainty around the mode estimate can be easily numerically summarized, for example using Bayes factors against neighbouring model configurations.

An explanation of how to obtain default values for the prior hyperparameters and a description of the greedy algorithm can be found in Supplementary Text S1.

### Data collection

Data collection followed a multi-stage approach. First, 12 295 A/H3N2 HA protein sequences were downloaded from three different search engines: NCBI's Influenza Virus Resource (Bao *et al.*, 2008), GISAID EpiFlu Database (Bogner *et al.*, 2006) and Influenza Research Database (Squires *et al.*, 2012). Our search query consisted of full-length A/H3N2 HA proteins, collected from human hosts in any country, excluding laboratory strains and mixed subtypes. In the second stage, we scanned the data for duplicates and low-quality reads and, after removing them from the collection, we aligned the data using muscle (Edgar, 2004). After again removing duplicates, the dataset consisted of 4898 unique strains of 567 amino acids. The complete list of accession numbers is given in Table S1.

### 3-D mapping of characteristic amino acid changes

The amino acid positions that correspond to characteristic amino acid changes were mapped to the crystal structure of the influenza virus HA (PDB ID 1HA0). Structurally relevant mutations occurring between two consecutive clusters are shown as yellow spheres. The resulting sequence of mapping images were rendered in PyMol and the image sequence was then encoded into a video file using MEncoder v.4.8.3 and the H.264 compression format.

### Broad overview of K-Pax2 output

To obtain a reliable estimate of the model parameters, we ran the optimization algorithm 100 times from different starting points and chose the solution with the highest posterior probability. The starting points were created by randomly modifying, through merging and splitting operations, a common *k*-medoids partition (Hastie *et al.*, 2009). The value for *k* was chosen according to the highest posterior probability score. This procedure generated initial partitions lying in a neighbourhood of the optimal solution and allowed the algorithm to converge in less than 6 h (2.6 GHz processor with 2 GB RAM). The optimal model allocated the 4898 sequences into 57 different groups while simultaneously detecting 117 (out of 567 possible) cluster-defining sites. As a comparison, the adjusted Rand index between our solution and the *k*-medoids partition with the same number of clusters is 0.824. The two partitions are very similar and their discrepancy is completely explained by a small rearrangement of the units. This result can be interpreted by noting that Kpax2 gives different weights to matrix columns, whereas standard clustering techniques do not make any distinction between noise and signal sites.

**Fig. 1.**
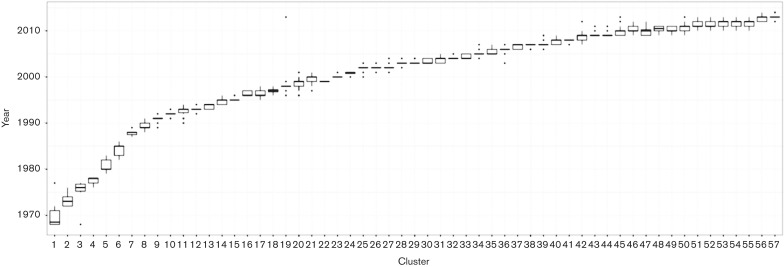
Temporal distribution of influenza A/H3N2 HA within each K-Pax2 cluster. Groups are sorted by sampling year of the earliest consensus sequence.

**Fig. 2.**
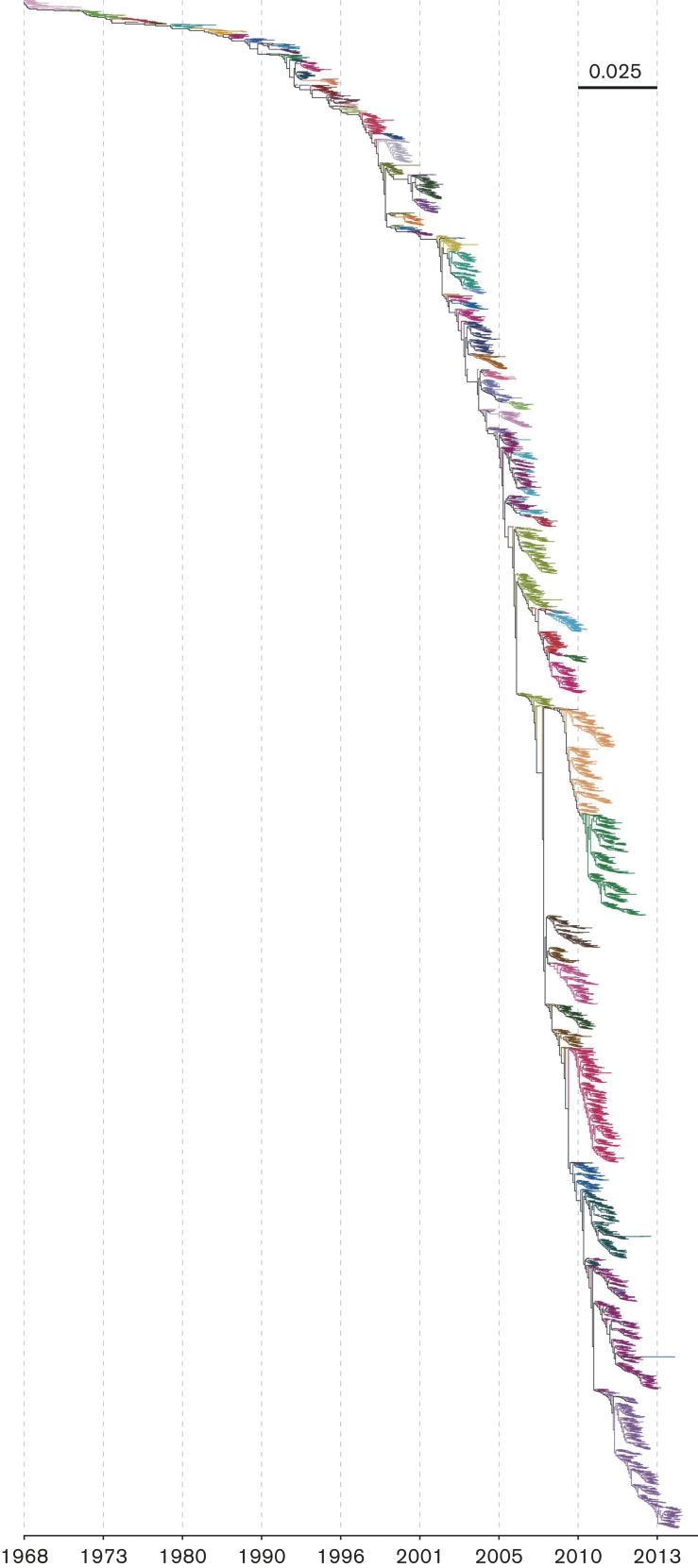
Maximum-likelihood phylogenetic tree of influenza A/H3N2 HA. K-Pax2 clusters are denoted in the tree as different colours. The scale bar indicates the expected number of substitutions per site.

To understand the groups’ chronologies, we first selected, within each group, only those strains possessing the whole set of characteristic amino acids. We will call these strains the ‘consensus sequences’ of the cluster, as they represent the molecular variation most relevant for selection. Based on the earliest year in which the consensus sequence was identified, we ordered the groups according to their appearance. Fig. 1 summarizes the temporal distribution within each cluster, showing a clear relationship between cluster associations and sampling time. A similar temporal pattern can be observed by overlaying the clusters on a maximum-likelihood phylogenetic tree (Fig. 2). Because more samples are available from the recent past, we achieve higher resolution clustering of samples from the past several years compared with, for example, samples from 1968 to 1972.

As shown in Table S2, each virus group is associated with a particular subset of the 117 sites. The cluster-defining amino acids can be interpreted as a fingerprint of the fitness change that did lead to proliferation of the lineage represented by the cluster.

### Core evolution of the HA protein

To facilitate comparison with HA evolution, we performed a phylogenetic analysis of the fingerprint amino acid change patterns discovered by the method. There can exist, in particular with densely sampled data from co-circulating groups of strains, multiple clusters of which only one successfully seeds the next cluster. Therefore, we identified a parsimonious ‘core’ set of groups defined to have the following characteristics. First, their age or time of emergence is determined by the first sampling date of their consensus sequence (as previously defined). Second, a core cluster can have only a single ancestral core cluster but potentially multiple descendant clusters, some of which may not be core clusters themselves. Third, a core cluster can descend only from an ancestral core cluster that precedes it by at least 1 year. In addition, we assumed that no recombination has occurred.

**Fig. 3.**
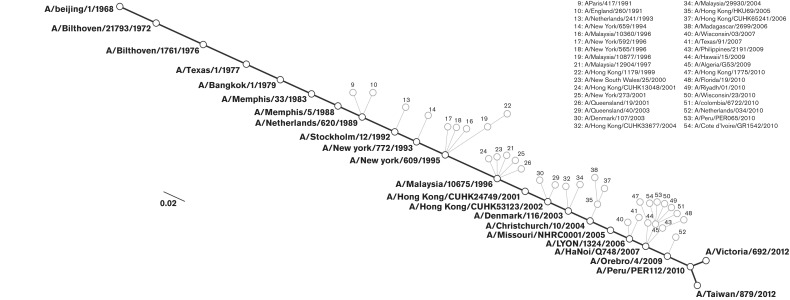
Phylogeny of influenza A/H3N2 HA as a phylogeny of K-Pax2 clusters. Ancestors are defined as the minimum (average) genetic distance groups, at least 1 year older. Each cluster is labelled by its earliest consensus sequence. Highlighted clusters connecting the viruses observed in 1968 to the most recent ones are the ‘core’ clusters. The scale bar indicates the expected number of substitutions per site.

**Fig. 4.**
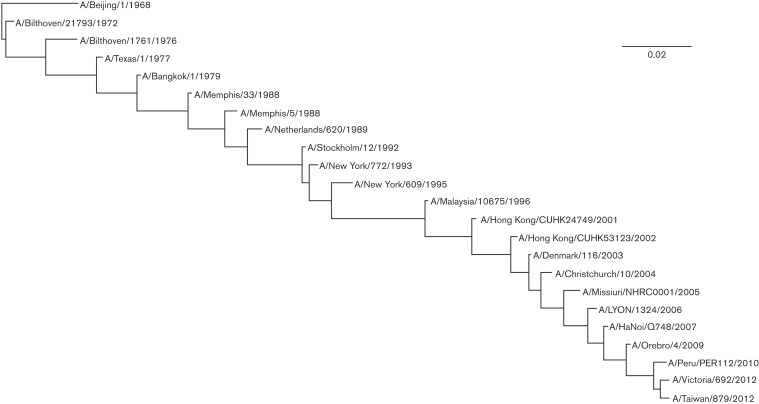
Maximum-likelihood phylogenetic tree of influenza A/H3N2 HA, restricted to core cluster consensus sequences. The 23 strains are the core clusters’ earliest consensus sequences. The scale bar indicates the expected number of substitutions per site.

The above criteria led to the discovery of 23 core clusters among the 57 clusters present in the K-Pax2 output. We computed the genetic distance between clusters as the average distance between their consensus sequences using the corrected distance proposed by Tamura & Kumar (2002) and the usual p-distance (Nei & Kumar, 2000). Both measures agreed. The tree in Fig. 3 was reconstructed by choosing, for each group, the ancestor associated with the minimum distance. The core clusters can be interpreted as the backbone clades of the A/H3N2 HA phylogeny, connecting the viruses observed in 1968 to the most recent ones. The classical ladder shape of the phylogenetic tree is conserved when only one consensus sequence per core cluster is used (Fig. 4). These cluster transitions closely resemble those reported by Smith *et al.* (2004) based on a carefully curated set of sequences, which represents less than 10 % of the data analysed here. The evolutionary relationships among all the 57 clusters are shown in Fig. S1.

**Fig. 5.**
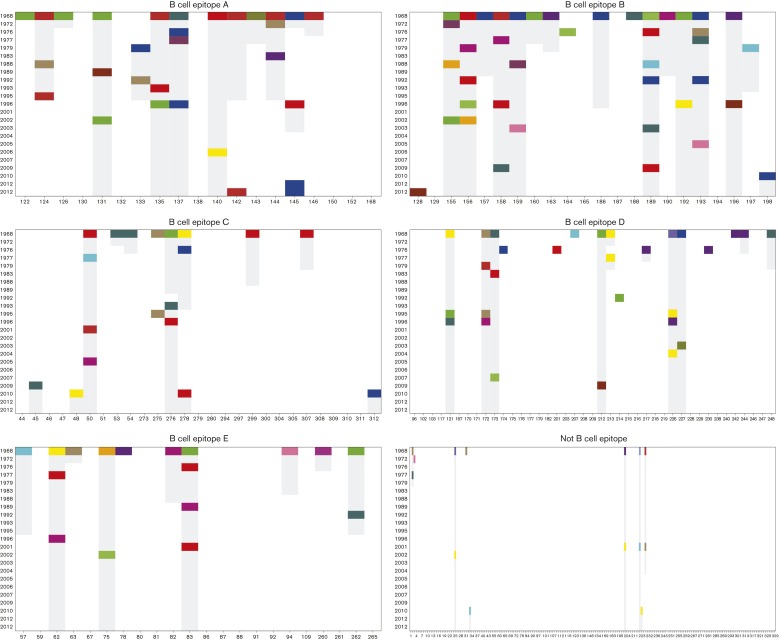
HA1 chain characteristic sites and their changes across the 23 core clusters. Vertical grey bars indicate cases where the previous characteristic amino acid in the sequence position has not mutated to a new value. White in any position indicates that the amino acid is not determined as characteristic. All other colours correspond to specific amino acids. Abscissae indicate residues' position along the HA protein.

Figure 5 shows how the characteristic sites of the core clusters have evolved over time. This reflects the dominant role of the B-cell epitopes in contrast to T-cell epitopes (Suzuki, 2006). To quantify the distribution of these changes over time, we calculated unadjusted estimates of mutation rates in each epitope and elsewhere in HA1 (Table 1). Antigenic drift is thought to occur when an average of four amino acid changes accumulates over time (Koel *et al.*, 2013). Many of the cluster transitions in Fig. 5 agree with this definition, but some carry fewer substitutions, which illustrates the usefulness of more flexible, statistical model-based rules to pinpoint potential targets for further attention and experimental work. The inferred changes are not uniformly distributed over the five epitopes *(chi*-squared test, χ^2^ = 19.665, df = 4, *P* < 0.001); instead changes in epitopes B and A are over-represented (in decreasing order), which matches well with current understanding of their relative functional importance (Koel *et al.*, 2013).

**Table 1:** Unadjusted mutation rate estimates, as observed on the HA1 of influenza A/H3N2, by B cell epitope (BCE) Rates have been estimated as , where  is the total number of amino acid changes, *l* is the length of the region and *t* is the time difference in years between two clusters. Independence between sites and homogeneous rates per region are assumed.

Year	A	B	C	D	E	Not BCE	HA1 global
1972	0.0263	0.0227	0.0093	0.0122	0.0114	0.0025	0.0076
1976	0.0395	0.0341	0.0185	0.0305	0.0227	0.0013	0.0121
1977	0.1053	0.1364	0.0741	0.122	0.0909	0.005	0.0455
1979	0.0526	0.0682	0	0.0244	0	0	0.0106
1983	0.0132	0.0114	0.0093	0.0183	0	0.0013	0.0053
1988	0.0105	0.0273	0	0	0.0091	0	0.003
1989	0.0526	0	0.037	0	0.0909	0	0.0121
1992	0.0175	0.0758	0.0123	0.0081	0.0152	0	0.0091
1993	0.0526	0	0	0.0244	0	0	0.0061
1995	0.0263	0.0227	0.037	0.0366	0	0	0.0106
1996	0.3684	0.1818	0.0741	0.0732	0.1364	0	0.0576
2001	0	0.0091	0.0074	0	0.0091	0.003	0.0036
2002	0.0526	0.0909	0	0	0.0455	0.005	0.0152
2003	0	0.0909	0	0.0244	0	0	0.0091
2004	0.0526	0	0	0.0244	0	0	0.0061
2005	0	0.0455	0.037	0	0	0.005	0.0091
2006	0.0526	0	0	0	0	0	0.003
2007	0	0	0	0.0244	0	0	0.003
2009	0	0.0455	0.0185	0.0122	0	0	0.0061
2010	0	0.0455	0.1111	0	0	0.0101	0.0182
2012(a)*	0.0263	0	0	0	0	0	0.0015
2012(b)*	0.0526	0.0227	0	0	0	0	0.0045
Global†	0.0311	0.0351	0.0152	0.0161	0.0145	0.0014	0.0092

* Mutations since 2010.

† It is unknown which of the two 2012 co-circulating groups will go extinct. The global rate has been computed by arbitrarily choosing cluster 2012(a).

**Fig. 6.**
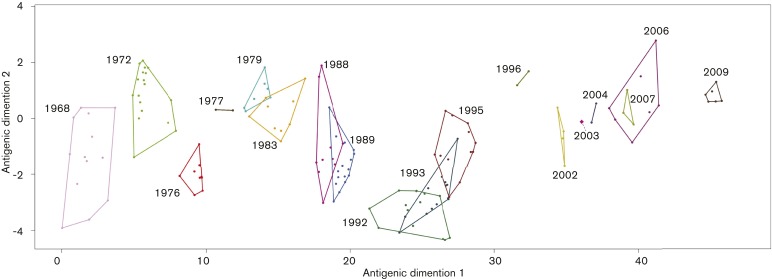
Core clusters in antigenic space. Polygon shapes and sizes are dependent on the availability of inhibition assay data.

Figure 6 shows how the core clusters relate to each other in antigenic space, based on haemagglutination inhibition assays (Bedford *et al.*, 2014). Many clusters are clearly distinct from each other, supporting the conclusion that K-Pax2 successfully identifies meaningful phenotypes. The pairs of clusters where an overlap occurs represent core clusters that arise in succession in Fig. 4. This suggests that our method has high sensitivity to detect changes that relate to early antigenic separation of strains, making it potentially also useful for continuous semi-automated screening of novel antigenic types from sequenced strains.

While K-Pax2 can generate hypotheses about which amino acids are under selection simply on the basis of sequence data, the integration of K-Pax2 output and other data can yield additional hypotheses. Video S1 displays the characteristic amino acid changes in core clusters mapped to the 3D structure of HA. The most comprehensive transition occurs in the 1996 group where changes occurred in all five epitopes, shown as a pronounced jump in antigenic space (Fig. 6). Interestingly, sequential changes in core characteristic sites rarely occur in close proximity, even when within the same epitope. This raises the possibility that selection tends to favour alternation across the protein surface, even within a single domain. Such patterns are consistent with the idea that HA evades immunity through sequential mutations that enable escape from different subpopulations (Linderman *et al.*, 2014; Sato *et al.*, 2004).

## Conclusions

There is a widening gap between the number of experimentally validated evolutionary mechanisms and the abundance of sequence data. Hence, there is demand for computational tools that can aid in harvesting biologically meaningful signals from data to guide further research.

Using thousands of publicly available HA sequences from A/H3N2 since 1968, we demonstrated that a Bayesian modelling approach can identify patterns of sequence variation that reflect known existing drivers of A/H3N2 evolution. These results suggest the power of K-Pax2 to extract evolutionary signals from microbial sequence collections and to provide a critically needed tool to guide studies of protein function and evolution.

Despite the demonstrated ability of our method to successfully explore sequence variation without imposing an explicit dynamic evolutionary model, there are caveats to be aware of. Like most statistical methods, the model-based clustering can be affected by sampling biases of various kinds. Highly uneven sampling over space and time will both reduce the power to detect novel variants and inflate the false positive rate of functionally critical residue changes. Furthermore, certain evolutionary processes such as episodic selection can create a pattern that resembles those implied by positive selection, and hence the inferred clusters may lack meaningful interpretation in phenotype space. Furthermore, hitchhiking phenomena due to genetic linkage may confound the identification of the causal variants as characteristic sites.

K-Pax2 has been implemented as an R package and is freely available at http://www.helsinki.fi/bsg/software/kpax2/ and at https://github.com/alberto-p/kpax2.

## Acknowledgements

We acknowledge the authors, and originating and submitting laboratories of the sequences from GISAID EpiFlu Database on which this research is based (the list is detailed in Table S3). We thank Trevor Bedford for kindly providing us with the data points used to create Fig. 6.

## References

[mgen000025-Aguas1] Aguas R. Ferguson N.M. (2013). Feature selection methods for identifying genetic determinants of host species in RNA viruses PLOS Comput Biol 9 e1003254 10.1371/journal.pcbi.1003254. 24130470 PMC3794897

[mgen000025-Bao1] Bao Y. Bolotov P. Dernovoy D. Kiryutin B. Zaslavsky L. Tatusova T. Ostell J. Lipman D. (2008). The influenza virus resource at the National Center for Biotechnology Information J Virol 82 596–601 10.1128/JVI.02005-07. 17942553 PMC2224563

[mgen000025-Bedford1] Bedford T. Suchard M.A. Lemey P. Dudas G. Gregory V. Hay A.J. McCauley J.W. Russell C.A. Smith D.J. Rambaut A. (2014). Integrating influenza antigenic dynamics with molecular evolution eLife 3 e01914 10.7554/eLife.01914. 24497547 PMC3909918

[mgen000025-Benson1] Benson D.A. Karsch-Mizrachi I. Lipman D.J. Ostell J. Wheeler D.L. (2005). GenBank Nucleic Acids Res 33 D34–D38 10.1093/nar/gki063. 15608212 PMC540017

[mgen000025-Bernardo1] Bernardo J.M. Smith A.F.M. (2000). Bayesian Theory Chichester Wiley.

[mgen000025-Bizebard1] Bizebard T. Gigant B. Rigolet P. Rasmussen B. Diat O. Bösecke P. Wharton S.A. Skehel J.J. Knossow M. (1995). Structure of influenza virus haemagglutinin complexed with a neutralizing antibody Nature 376 92–94 10.1038/376092a0. 7596443

[mgen000025-Bogner1] Bogner P. Capua I. Lipman D.J. Cox N.J. other authors (2006). A global initiative on sharing avian flu data Nature 442 981–981 10.1038/442981a.

[mgen000025-Cheng1] Cheng L. Connor T.R. Sirén J. Aanensen D.M. Corander J. (2013). Hierarchical and spatially explicit clustering of DNA sequences with BAPS software Mol Biol Evol 30 1224–1228 10.1093/molbev/mst028. 23408797 PMC3670731

[mgen000025-Cotten1] Cotten M. Watson S.J. Kellam P. Al-Rabeeah A.A. Makhdoom H.Q. Assiri A. Al-Tawfiq J.A. Alhakeem R.F. Madani H. other authors (2013). Transmission and evolution of the Middle East respiratory syndrome coronavirus in Saudi Arabia: a descriptive genomic study Lancet 382 1993–2002 10.1016/S0140-6736(13)61887-5. 24055451 PMC3898949

[mgen000025-Edgar1] Edgar R.C. (2004). muscle: multiple sequence alignment with high accuracy and high throughput Nucleic Acids Res 32 1792–1797 10.1093/nar/gkh340. 15034147 PMC390337

[mgen000025-Fitch1] Fitch W.M. Leiter J.M. Li X.Q. Palese P. (1991). Positive Darwinian evolution in human influenza A viruses Proc Natl Acad Sci U S A 88 4270–4274 10.1073/pnas.88.10.4270. 1840695 PMC51640

[mgen000025-Fleury1] Fleury D. Barrère B. Bizebard T. Daniels R.S. Skehel J.J. Knossow M. (1999). A complex of influenza hemagglutinin with a neutralizing antibody that binds outside the virus receptor binding site Nat Struct Biol 6 530–534 10.1038/9299. 10360354

[mgen000025-Gire1] Gire S.K. Goba A. Andersen K.G. Sealfon R.S.G. Park D.J. Kanneh L. Jalloh S. Momoh M. Fullah M. other authors (2014). Genomic surveillance elucidates Ebola virus origin and transmission during the 2014 outbreak Science 345 1369–1372 10.1126/science.1259657. 25214632 PMC4431643

[mgen000025-Gong1] Gong L.I. Bloom J.D. (2014). Epistatically interacting substitutions are enriched during adaptive protein evolution PLoS Genet 10 e1004328 10.1371/journal.pgen.1004328. 24811236 PMC4014419

[mgen000025-Hastie1] Hastie T. Tibshirani R. Friedman J. (2009). The Elements of Statistical Learning 2nd edn. Berlin:10.1007/978-0-387-84858-7 Springer.

[mgen000025-Jain1] Jain S. Neal R.M. (2007). Splitting and merging components of a nonconjugate Dirichlet process mixture model Bayesian Anal 2 445–472 10.1214/07-BA219.

[mgen000025-Knossow1] Knossow M. Gaudier M. Douglas A. Barrère B. Bizebard T. Barbey C. Gigant B. Skehel J.J. (2002). Mechanism of neutralization of influenza virus infectivity by antibodies Virology 302 294–298 10.1006/viro.2002.1625. 12441073

[mgen000025-Koel1] Koel B.F. Burke D.F. Bestebroer T.M. van der Vliet S. Zondag G.C.M. Vervaet G. Skepner E. Lewis N.S. Spronken M.I.J. other authors (2013). Substitutions near the receptor binding site determine major antigenic change during influenza virus evolution Science 342 976–979 10.1126/science.1244730. 24264991

[mgen000025-Koser1] Köser C.U. Ellington M.J. Cartwright E.J.P. Gillespie S.H. Brown N.M. Farrington M. Holden M.T.G. Dougan G. Bentley S.D. other authors (2012). Routine use of microbial whole genome sequencing in diagnostic and public health microbiology PLoS Pathog 8 e1002824 10.1371/journal.ppat.1002824. 22876174 PMC3410874

[mgen000025-Linderman1] Linderman S.L. Chambers B.S. Zost S.J. Parkhouse K. Li Y. Herrmann C. Ellebedy A.H. Carter D.M. Andrews S.F. other authors (2014). Potential antigenic explanation for atypical H1N1 infections among middle-aged adults during the 2013–2014 influenza season Proc Natl Acad Sci U S A 111 15798–15803 10.1073/pnas.1409171111. 25331901 PMC4226110

[mgen000025-Marttinen1] Marttinen P. Corander J. Törönen P. Holm L. (2006). Bayesian search of functionally divergent protein subgroups and their function specific residues Bioinformatics 22 2466–2474 10.1093/bioinformatics/btl411. 16870932

[mgen000025-Marttinen2] Marttinen P. Myllykangas S. Corander J. (2009). Bayesian clustering and feature selection for cancer tissue samples BMC Bioinformatics 10 90 10.1186/1471-2105-10-90. 19296858 PMC2679022

[mgen000025-Meroz1] Meroz D. Yoon S.-W. Ducatez M.F. Fabrizio T.P. Webby R.J. Hertz T. Ben-Tal N. (2011). Putative amino acid determinants of the emergence of the 2009 influenza A (H1N1) virus in the human population Proc Natl Acad Sci U S A 108 13522–13527 10.1073/pnas.1014854108. 21808039 PMC3158228

[mgen000025-Neal1] Neal R.M. (2000). Markov chain sampling methods for Dirichlet process mixture models J Comput Graph Stat 9 249–265.

[mgen000025-Nei1] Nei M. Kumar S. (2000). Molecular Evolution and Phylogenetics Oxford Oxford University Press.

[mgen000025-Reuter1] Reuter S. Ellington M.J. Cartwright E.J.P. Köser C.U. Török M.E. Gouliouris T. Harris S.R. Brown N.M. Holden M.T.G. other authors (2013). Rapid bacterial whole-genome sequencing to enhance diagnostic and public health microbiology JAMA Intern Med 173 1397–1404 10.1001/jamainternmed.2013.7734. 23857503 PMC4001082

[mgen000025-Sato1] Sato K. Morishita T. Nobusawa E. Tonegawa K. Sakae K. Nakajima S. Nakajima K. (2004). Amino-acid change on the antigenic region B1 of H3 haemagglutinin may be a trigger for the emergence of drift strain of influenza A virus Epidemiol Infect 132 399–406 10.1017/S0950268803001821. 15188708 PMC2870118

[mgen000025-Skehel1] Skehel J.J. Wiley D.C. (2000). Receptor binding and membrane fusion in virus entry: the influenza hemagglutinin Annu Rev Biochem 69 531–569 10.1146/annurev.biochem.69.1.531. 10966468

[mgen000025-Smith1] Smith D.J. Lapedes A.S. de Jong J.C. Bestebroer T.M. Rimmelzwaan G.F. Osterhaus A.D.M.E. Fouchier R.A.M. (2004). Mapping the antigenic and genetic evolution of influenza virus Science 305 371–376 10.1126/science.1097211. 15218094

[mgen000025-Squires1] Squires R.B. Noronha J. Hunt V. García-Sastre A. Macken C. Baumgarth N. Suarez D. Pickett B.E. Zhang Y. other authors (2012). Influenza research database: an integrated bioinformatics resource for influenza research and surveillance Influenza Other Respi Viruses 6 404–416 10.1111/j.1750-2659.2011.00331.x. 22260278 PMC3345175

[mgen000025-Suzuki1] Suzuki Y. (2006). Natural selection on the influenza virus genome Mol Biol Evol 23 1902–1911 10.1093/molbev/msl050. 16818477

[mgen000025-Suzuki2] Suzuki Y. (2011). Positive selection for gains of N-linked glycosylation sites in hemagglutinin during evolution of H3N2 human influenza A virus Genes Genet Syst 86 287–294 10.1266/ggs.86.287. 22362027

[mgen000025-Tamura1] Tamura K. Kumar S. (2002). Evolutionary distance estimation under heterogeneous substitution pattern among lineages Mol Biol Evol 19 1727–1736 10.1093/oxfordjournals.molbev.a003995. 12270899

[mgen000025-Wolf1] Wolf Y.I. Viboud C. Holmes E.C. Koonin E.V. Lipman D.J. (2006). Long intervals of stasis punctuated by bursts of positive selection in the seasonal evolution of influenza A virus Biol Direct 1 34 10.1186/1745-6150-1-34. 17067369 PMC1647279

[mgen000025-Worobey1] Worobey M. Han G.-Z. Rambaut A. (2014). A synchronized global sweep of the internal genes of modern avian influenza virus Nature 508 254–257 10.1038/nature13016. 24531761 PMC4098125

